# A High-Phosphogypsum Multi-Solid-Waste Cementitious Binder for Backfill: Cross-Scale Insight into Pore Structure and Strength Development

**DOI:** 10.3390/ma19102156

**Published:** 2026-05-21

**Authors:** Jianhua Hu, Xingjian Jiang, Fengwen Zhao, Zhi Yu, Ying Zhou, Dehua Wang

**Affiliations:** 1 Zijin School of Geology and Mining, Fuzhou University, Fuzhou 350108, China; hujh21@fzu.edu.cn (J.H.); 231620003@fzu.edu.cn (X.J.); 2 Jiangxi Copper Technology Institute Co., Ltd., Nanchang 330096, China; dr_zfw94@163.com; 3 Hubei Sanning Mining Co., Ltd., Yichang 443100, China; yingzhou0410@163.com (Y.Z.); yjkzjpo29@163.com (D.W.)

**Keywords:** backfill material, phosphogypsum, industrial solid waste, pore

## Abstract

**Highlights:**

**What are the main findings?**

- A binder containing 40 wt.% Phosphogypsum (PG) was evaluated for cemented paste backfill.
- Strength development was interpreted using uniaxial compressive strength (UCS), pore-structure, microstructural, mineralogical, and image-derived pore metrics.
- AFt and C–S–H gels were associated with pore refinement.
- Pore probability entropy was most sensitive to 7-day UCS.

**What are the implications of the main findings?**

- The tested high-PG binder showed potential for targeted mine backfill.
- The combined pore structure and UCS analysis clarified micro–macro relationships.
- Image-based sensitivity analysis helps identify key pore parameters controlling strength.
- The results support the design of high-PG clinker-free binders.
- The findings support PG reuse in targeted phosphate-mine backfill.

**Abstract:**

Phosphogypsum (PG) is an industrial solid waste whose use in cementitious materials is limited by strength reduction at high dosages. This study evaluated a clinker-free multi-solid-waste binder containing 40 wt.% PG for cemented paste backfill using steel slag powder (SSP) and granulated blast-furnace slag (GBFS) as co-binders, with phosphate mine tailings and slime as aggregates. Uniaxial compressive strength (UCS), X-ray diffraction, scanning electron microscopy, and nuclear magnetic resonance were combined with image-based pore-structure sensitivity analysis to examine the relationships among hydration products, pore evolution, and strength development. The results showed that AFt and C–S–H-like gels were associated with pore refinement and strength gain. All mixtures reached UCS values above 0.5 MPa at 7 days and 1.0 MPa at 28 days. The A2 mixture achieved the highest 7-day UCS of 0.8 MPa, whereas A1 showed the highest 28-day UCS of 1.6 MPa. Porosity, pore probability entropy, and fractal dimension were negatively correlated with UCS, with pore probability entropy showing the highest sensitivity to 7-day strength. These findings support the use of high-PG clinker-free binders for targeted phosphate-mine backfill.

## 1. Introduction

Phosphogypsum (PG) is a solid waste by-product associated with the production of phosphoric acid in the phosphate industry. Its main component is calcium sulfate dihydrate (CaSO_4_·2H_2_O), and it contains impurities such as fluoride, phosphorus, and organic matter. PG is characterized by low water content, high acidity, and toxicity [1,2]. Statistics show that for every ton of phosphoric acid produced, 4 to 5 tons of PG are generated [3]. In China, the annual production of PG is approximately 80 to 85 million tons, with a utilization rate of only around 40 wt.% [4]. Currently, the global stockpile of PG has exceeded 6 billion tons, with an annual increase of more than 300 million tons, making it a global issue. There is an urgent need for methods and technological processes capable of processing large quantities of PG [5].

In this context, mine backfill, which requires massive amounts of cementitious materials and aggregates, provides a practical pathway for large-volume PG utilization. The use of PG and materials such as cement clinker to prepare multi-component cement systems as cementitious materials has been widely studied [6,7,8]. Beyond partially replacing clinker, the preparation of cementitious materials from all industrial solid wastes by combining PG with other active solid wastes has become a research hotspot in the utilization of PG [9]. However, due to the intrinsic characteristics of PG—such as its high sulfate content and various impurities that may interfere with hydration—significant challenges remain. For example, Wu et al. used desulfurized gypsum, fluorogypsum, and PG, mixed with steel slag (SS) and slag to prepare cementitious materials, and found that the UCS of PG-based backfill was the lowest among all the groups [10]. Zhao et al. used PG, cement, and quicklime to prepare backfill material and found that as the PG content increased, the 3-day and 7-day uniaxial compressive strength (UCS) showed a decreasing trend, with the 28-day UCS reaching its maximum at a PG content of 20% [11]. Liu et al. investigated the incorporation of PG at dosages of 0–40 wt.% in ultra-high-performance concrete (UHPC) and confirmed that PG affects the development of key hydration products. Strength tests showed that a moderate PG replacement level (10–20%) can maintain or even improve the long-term mechanical performance, whereas excessive PG replacement has an adverse effect on strength development [12]. In conclusion, because excessive PG generally reduces UCS, the PG content in gypsum-based binders is usually limited to about 20%. However, large-volume PG utilization in mine backfill requires the exploration of high-PG binder systems. The behavior of relatively high PG contents in clinker-free SSP/GBFS-based backfill binders remains insufficiently understood, as previous studies have mainly focused on lower PG dosages or cement/lime-assisted or alkali-activated systems.

To explore the high-volume utilization of PG in cementitious materials, a clinker-free binder system was designed in which PG, steel slag powder (SSP) and granulated blast-furnace slag (GBFS) together constitute the cementitious components. Chen et al. kept the GBFS content constant at 60 wt.% and varied the SSP/PG ratio from 40/0 to 0/40, and reported that mixtures incorporating a moderate amount of PG (10–20 wt.%) together with SSP achieved much higher 28-day compressive strengths than the PG-free mix, whereas further increasing the PG content to 30–40 wt.% led to a sharp strength reduction, highlighting the synergy and the need for a proper balance among SSP, GBFS and PG [13].

Inspired by the above findings and the demand for high-PG binder systems, this study fixed the PG content at 40 wt.% of the binder, which is approximately twice the conventional upper limit. This dosage was selected as a deliberately challenging level to evaluate whether PG can be incorporated at a relatively high content into a clinker-free SSP/GBFS-based backfill binder while still meeting the strength requirement of the targeted phosphate mine. SSP and GBFS were used as co-binders to evaluate a high-phosphogypsum multi-solid-waste cementitious binder for backfill. Uniaxial compressive strength, X-ray diffraction, scanning electron microscopy, and nuclear magnetic resonance were combined to investigate hydration behavior, pore structure evolution, and strength development, thereby clarifying the relationship between hydration products, pore characteristics, and macroscopic mechanical performance.

## 2. Experimental Materials and Methods

### 2.1. Raw Materials

**Figure 1:**
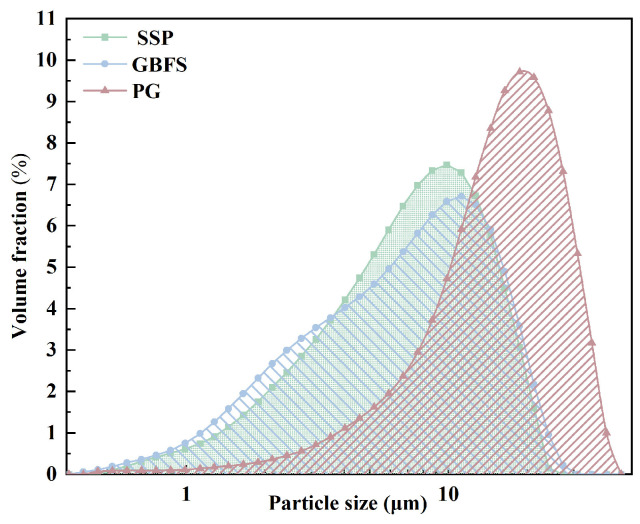
Size distribution of cementitious materials.

The aggregates used in the experiment were obtained from tailings and slime of a phosphate mine in Hubei Province. The PG in the cementitious materials came from the waste PG of a chemical enterprise upstream of the mine, while SSP and GBFS were sourced from waste slag materials of a steel plant. The water used in the experiment was ordinary tap water. SS was pre-ground in a planetary ball mill for ten minutes. The pretreated experimental materials were analyzed for their physical properties using sieving, laser particle size analysis, and X-ray fluorescence spectroscopy (XRF). The particle-size distribution of the fine cementitious materials was measured by the laser diffraction method after sufficient sample dispersion to minimize particle agglomeration. The chemical compositions of PG, SSP, and GBFS were determined by XRF using dried and finely ground powder samples. The particle size distribution is shown in Figure 1 and Table 1 and Table 2, while the chemical composition is presented in Table 3.

The collected PG was oven-dried at 50 °C and sieved to remove coarse impurities before use. To reduce compositional variability, all PG used in this study was taken from the same batch. The PG contains a relatively high SO_3_ content, together with small amounts of common impurities such as P_2_O_5_ and F^−^, which may influence ettringite formation and pore filling; this aspect will be discussed in Section 3.

### 2.2. Preparation of Backfill Samples

The PG content in the cementitious materials is set at 40 wt.% as the baseline value, with SSP and GBFS used as co-binders. The material proportions are shown in Table 4. According to the current aggregate mix in the backfill system used in the mine, the aggregates of the backfill consist of a 3:1 mixture of phosphate mine tailings and slime. The cementitious materials and aggregates are mixed at a mass concentration of 76%, with a binder-to-aggregate ratio of 1:4, to prepare the cemented paste backfill. These ratios were selected based on the existing backfill practice of the targeted phosphate mine and preliminary laboratory observations to ensure slurry homogeneity and stable specimen preparation.

Backfill Preparation and Curing Process: Cementitious materials with a fixed PG content of 40 wt.% were prepared according to the proportion shown in Table 4. First, PG, SSP and GBFS were dry-mixed to obtain a uniform binder, and then the binder was blended with tailings and slime in a mechanical mixer for several minutes; finally, water was added gradually until a homogeneous slurry without visible lumps was formed. The slurry was then evenly poured into cylindrical molds (*φ*50 mm × 100 mm) to prepare samples. The samples were labeled in sequence as A1–A4, with 6 samples per group, for a total of 24 samples. After 24 h in the molds, the samples were demolded and cured in a curing chamber at 20 °C and 95% humidity until the 7th and 28th days for the required mechanical and microstructural tests.

### 2.3. Test Methods

**Figure 2:**
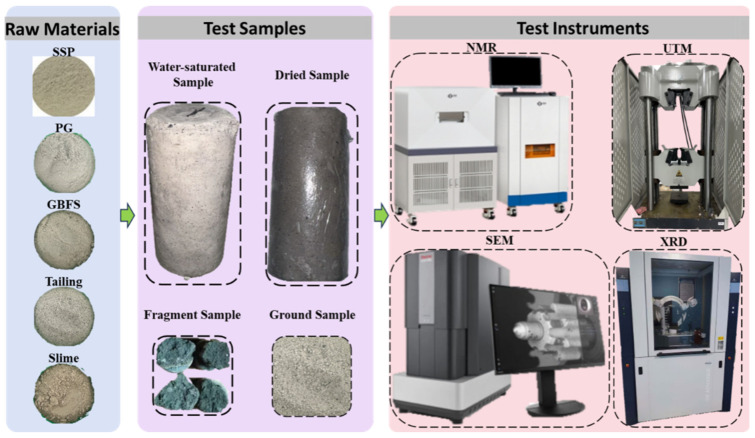
Test instruments and samples.

The test instruments and samples used in the study are shown in Figure 2 to provide visual information on the experimental setup and sample morphology, thereby improving the transparency and reproducibility of the testing procedure. At the macroscale, uniaxial compressive strength (UCS) tests were carried out on the backfill specimens, while at the microscale, nuclear magnetic resonance (NMR), scanning electron microscopy (SEM), and X-ray diffraction (XRD) tests were conducted. First, the samples were saturated before NMR testing. The samples were immersed in water for 24 h, and the surface water was gently removed before measurement. All samples were then tested immediately using the same NMR settings. The NMR test was conducted using an NMR instrument (MesoMR23-060H-I) manufactured by Niumai, Suzhou, China, to characterize the initial pore structure. Then, after natural drying, the samples were subjected to uniaxial compression tests on a WAW-1000D universal testing machine produced by Jinan Fangyuan Testing Instrument Co., Ltd.: Jinan, China, with a loading rate of 0.5 mm/min. Fragments taken from the central region of the failed specimens were dried, gold-sputtered, and observed under a scanning electron microscope (SEM) manufactured by JEOL (Tokyo, Japan) to examine the microstructure and phase assemblage. Finally, a small amount of crushed material was ground into powder, and the hydration products were analyzed using a D8 Advance X-ray diffractometer (XRD) produced by BRUKER (Karlsruhe, Germany).

In this study, the NMR and UCS data at different curing ages were obtained from three cylindrical samples prepared from the same batch of slurry for each mix, providing quantitative information on pore structure and strength. For the UCS test, three parallel cylindrical specimens were prepared for each mix and curing age. Because some specimens showed abnormally high deviations during testing, the maximum value among the three parallel specimens in each group was excluded using the same criterion, and the reported UCS value was calculated as the mean of the remaining two values. This conservative treatment was adopted to reduce the influence of anomalously high values on the strength evaluation. In contrast, SEM and XRD analyses were performed on a representative fragment taken from one sample of each mix at the target curing age. These procedures help ensure that the analysis of the micro–macro mechanisms of the high-PG backfill is as little affected by experimental errors as possible.

### 2.4. Use of Generative AI for Language Editing

During manuscript preparation, ChatGPT 5 was used only for language editing to improve grammar, clarity, and readability. It was not used to design the experiments, generate scientific content, analyze data, interpret results, prepare figures or tables, or formulate conclusions. All AI-assisted language revisions were reviewed and edited by the authors, who take full responsibility for the scientific content of the manuscript.

## 3. Results and Discussion

### 3.1. Microstructural Hydration Mechanism

**Figure 3:**
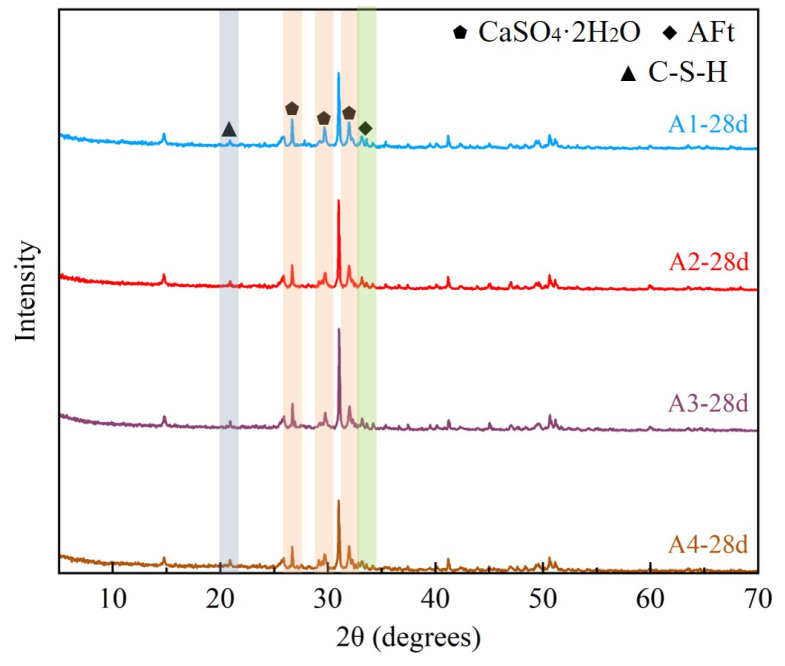
Phase composition of backfill samples at 28-day curing age.

The XRD test results of the backfill after 28 days of curing with a high PG content are shown in Figure 3. The major diffraction peaks were assigned to ettringite (AFt) and residual calcium sulfate dihydrate (CaSO_4_·2H_2_O), indicating that sulfate-bearing phases and AFt were the main crystalline phases detected after curing. Considering the low crystallinity of C–S–H, the XRD results were used mainly to identify crystalline phases, while the C–S–H-related interpretation was further supported by the SEM morphological observations discussed later in this section. Overall, the XRD patterns of A1–A4 were similar, which can be attributed to the fixed PG content of 40 wt.% and the use of the same raw materials in all mixtures. Therefore, changing the SSP/GBFS ratio did not produce new dominant crystalline phases, but mainly affected the relative intensity of the identified peaks. Compared with the groups containing lower SSP contents, the high-SSP groups showed relatively limited evidence of C–S–H formation when the XRD results were considered together with SEM observations, suggesting that the SSP/GBFS ratio mainly influenced the degree of hydration and the relative development of hydration products rather than changing the main crystalline phase assemblage. The cementitious material composition analysis revealed that the main components of GBFS include vitreous Calcium Silicate (CaSiO_3_), Calcium Aluminate (CaAl_2_O_4_), and a small amount of Iron Oxide (FeO/Fe_2_O_3_). In this binder system, no external alkaline activator was added. The alkaline condition was mainly generated internally by SSP hydration/dissolution, especially from free CaO and Ca-bearing silicate phases, which released Ca^2+^ and OH^−^ into the pore solution. Under this condition, GBFS can release reactive silica and alumina species, which participate in hydration reactions and contribute to the formation of AFt and C–S–H [13]. SSP mainly consists of Calcium Silicate (CaSiO_3_) and free Lime (f-CaO), and it provides Ca^2+^ and alkalinity for the hydration and activation reactions [14,15,16]. In the XRD patterns, no obvious diffraction peaks of alkaline crystalline phases, such as portlandite Ca(OH)_2_ or residual free CaO, were detected at 28 days. This suggests that the alkaline components supplied by SSP did not remain as detectable crystalline phases after hydration. Since XRD identifies crystalline phases rather than free ions, this result was not used to directly determine the OH^−^ concentration in the pore solution. Nevertheless, when considered together with the limited C–S–H formation observed in the high-SSP groups, this may indirectly suggest the relatively low reactivity of SSP in the high-PG binder system, which is consistent with previous reports that steel slag shows weaker early activation than more reactive slag-based components [17]. PG’s main component is CaSO_4_·2H_2_O, which generates SO_4_^2−^ ions during hydration, providing an environment for the formation of AFt [18]. The XRD results show that after 28 days of curing, a large amount of CaSO_4_·2H_2_O remains in the backfill, suggesting the persistence of sulfate-bearing phases in the high-PG system.

**Figure 4:**
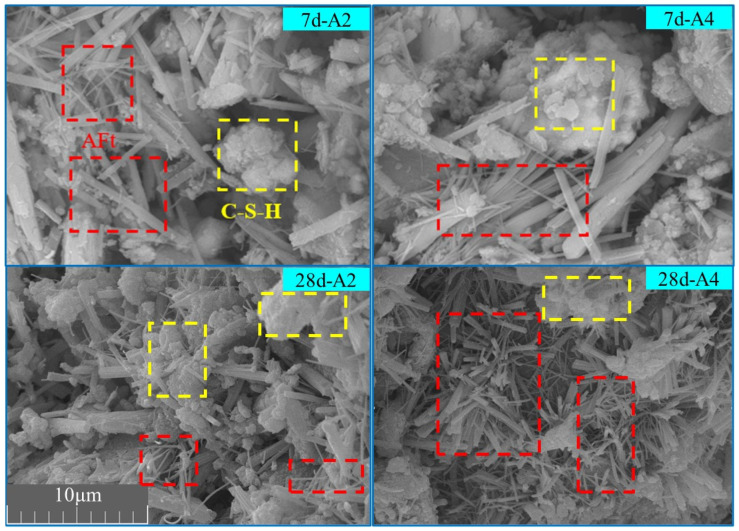
SEM images of different groups of backfill at various curing ages.

By using SEM to magnify the surface morphology of fragments at 5000× magnification for samples at different curing ages, the microstructural features of the high-PG cemented paste backfill were analyzed. The morphology and arrangement of the hydration products inside the backfill were examined, yielding the results shown in Figure 4. All SEM images were acquired at the same magnification and share the same scale; therefore, the scale bar shown in the figure applies to all panels. As shown in Figure 4, needle-like AFt crystals can be observed in the samples. In addition, some amorphous gel-like hydration products were observed, which may be associated with C–S–H-type gels according to their morphology, XRD results, and previous studies on slag-based cementitious systems. However, SEM observations alone cannot uniquely identify or quantify C–S–H; therefore, the discussion of C–S–H-related products in this study is qualitative [14]. Therefore, the main hydration reaction equation for the high-PG cemented paste backfill is as follows:

(1) $$2AlO_{4}^{5-}+6SO_{4}^{2-}+12Ca^{2+}+2OH^{-}+61H_{2}O\to 2AFt$$

(2) $$SiO_{4}^{4-}+xCa^{2+}+yH_{2}O\to C-S-H$$

Based on the above XRD and SEM analyses, the phase development in the high-PG binder system can be interpreted from the complementary chemical roles of PG, SSP, and GBFS. PG supplies sulfate-bearing phases, SSP provides Ca-bearing and alkaline components, and GBFS contributes reactive silicate and aluminate species. Accordingly, Equations (1) and (2) represent the principal chemical pathways leading to AFt and C–S–H-related gel products. This interpretation is consistent with the XRD identification of AFt and residual CaSO_4_·2H_2_O, as well as the SEM observation of needle-like crystals and gel-like products.

The SEM images at different curing ages reveal the temporal and spatial characteristics of the hydration reaction. At 7 days, the main hydration product in the backfill was needle-like AFt, with relatively few C–S–H gels. In the A4 group, which had a higher SSP content, the amount of C–S–H was significantly lower than in the A2 group, with only a small amount of C–S–H present around the AFt. At 28 days, in the A2 group, C–S–H and AFt formed a stable structure together, while in the A4 group, the hydration products were still predominantly AFt, with larger pores between the different hydration products. Considering the high solid-waste content of the binder, some unreacted raw-material particles may still remain in the matrix; therefore, isolated angular particles were not directly assigned to hydration products. There are two reasons for this hydration reaction: On one hand, the large accumulation of AFt in the early stages reduced the contact area between the aggregates and water, hindering further hydration. On the other hand, the lower activity of SSP led to insufficient active Ca^2+^ and OH^−^ ions, and with the high PG content, excess SO_4_^2−^ inhibited the progress of reaction (2). Therefore, reaction (1) plays a dominant role in the overall hydration reaction, while reaction (2) occurs less frequently, which is consistent with the results obtained from XRD phase analysis.

Nuclear Magnetic Resonance (NMR) can be used to assess the microporosity, pore distribution, and other microparameters of the backfill. There is a linear functional relationship between the T_2_ value and pore size, as shown in Equation (3) [19]:

(3) $$r=\rho_{2}F_{S}T_{2}$$

In the equation, *r* represents the pore radius in μm, and *T*_2_ represents the transverse relaxation time in ms. C, $\rho_{2}$, and $F_{S}$ are constants, where $\rho_{2}$ represents the transverse surface relaxation intensity in μm/ms, and $F_{S}$ represents the pore shape.

**Figure 5:**
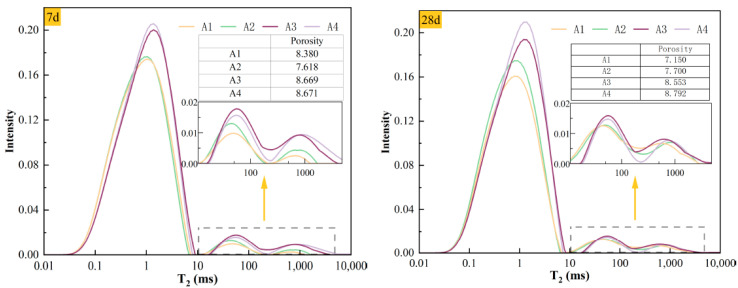
*T*_2_ empirical spectra of different groups of backfill at various curing ages.

The *T*_2_ relaxation time spectra and porosity test results for different curing ages of each group are shown in Figure 5. The porosity values obtained from NMR were also included in Figure 5 to assist the interpretation of the *T*_2_ spectra, because peak intensity alone does not fully represent the integrated pore signal. To reduce the influence of possible environmental and instrument variations between different testing batches, the porosity values were mainly compared within the same curing age. At 7 days, A2 showed the lowest porosity of 7.618%, indicating better early pore refinement than the other mixtures. At 28 days, A1 showed the lowest porosity of 7.150%, while A4 remained the highest at 8.792%, suggesting more favorable later-age densification in A1. These results indicate that A2 had a better early-age pore structure and strength response, whereas A1 exhibited better later-age pore densification. Therefore, the NMR results were interpreted together with UCS, SEM observations, and image-based pore metrics rather than used alone to define the optimal mixture. In the *T*_2_ relaxation time distribution spectra, the x-axis represents the duration of the *T*_2_ relaxation time, which is positively correlated with pore size. That is, the peaks on the right side of the spectra correspond to larger pore sizes, while the y-axis amplitude reflects the relative abundance of the corresponding pore structures. A higher amplitude indicates a larger proportion of pores of that size. To accurately describe the pore sizes corresponding to different peaks, this section follows empirical ranges reported in previous studies: pores with T_2_ < 10 ms are defined as micropores, those with 10–100 ms are defined as mesopores, and those with T_2_ > 100 ms are defined as macropores [20,21]. As shown in Figure 5, the *T*_2_ spectra of all groups at different curing ages exhibit three distinct peaks, with the peak values decreasing from left to right. This indicates that the high-PG cemented paste backfill contains three types of pores with different diameters, the pore structure is dominated by micropores, followed by mesopores, with only a small fraction of macropores. At 7 days, all three peak values increase with the SSP content. At 28 days, the increase is mainly in the micropores with higher SSP content. This is due to the incomplete hydration reaction at 7 days, resulting in a loose microstructure of the high-PG cemented paste backfill. By 28 days, the hydration reaction is nearly complete, and the hydration products have filled the larger pores [22,23].

**Figure 6:**
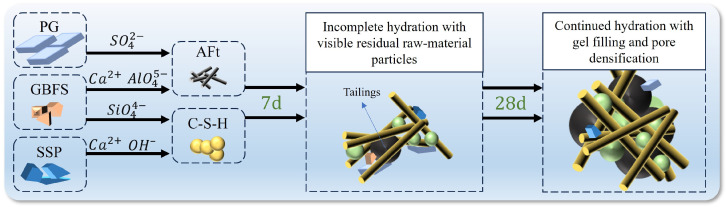
Hydration Process and Mechanism.

In conclusion, in the high-PG cemented paste backfill, GBFS provides active alumino-silicate tetrahedral structures, PG provides a large amount of SO_4_^2−^, and SSP offers an alkaline environment and some necessary ions for the reaction. The mixture of these three materials as cementitious materials can synergistically undergo hydration. A schematic diagram of the hydration mechanism is shown in Figure 6. In the early stages, the abundant SO_4_^2−^ provided by high PG, combined with the rapid hydration of SSP to generate OH^−^, Ca^2+^, and Al^3+^, reacts with SO_4_^2−^ to form needle-like AFt (as shown in Equation (1)), ensuring early binding of the backfill. At the same time, the alumino-silicate tetrahedral structures in the GBFS decompose, and the broken Al-O and Si-O bonds react with other hydrolyzed ions to form AFt and C–S–H (as shown in Equations (1) and (2)), ensuring the continuous generation of hydration products. However, due to the excess SO_4_^2−^ provided by high PG and the low activity of SSP leading to insufficient OH^−^, the formation of AFt remains dominant throughout the entire hydration period. After the hydration reactions have fully progressed, on the microstructural level, AFt plays a supporting role in the backfill, while C–S–H acts as a filler, reducing the pores between different particles and together forming a more compact structure.

### 3.2. Macroscopic Mechanical Properties

**Figure 7:**
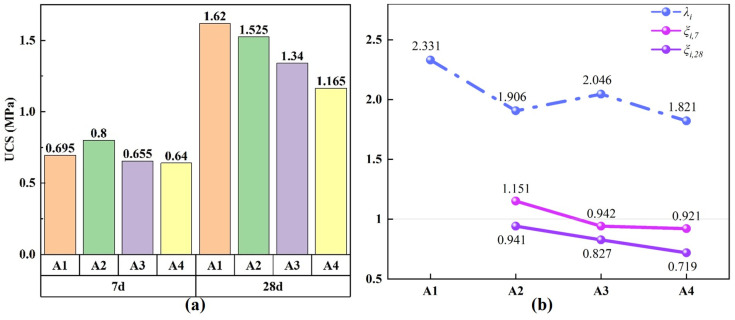
Mechanical properties of backfill samples: (**a**) UCS at different curing ages; (**b**) strength enhancement coefficients.

**Figure 8:**
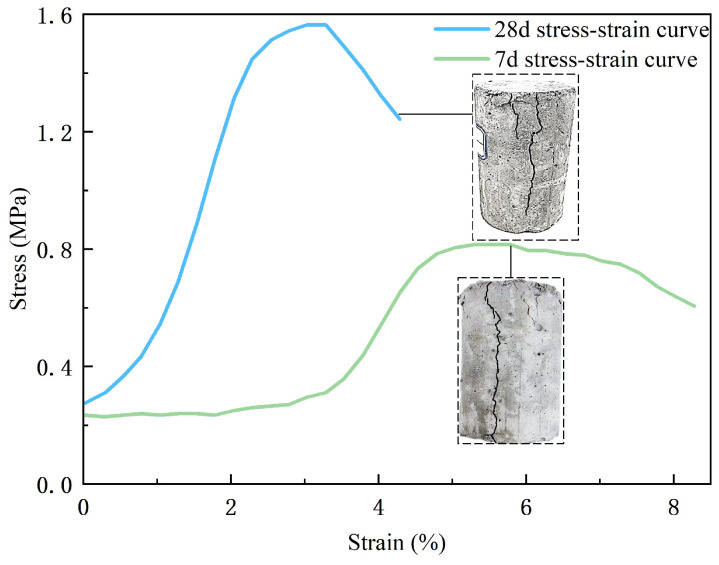
Failure stress–strain curves and images of failed samples for A2.

Uniaxial compressive strength (UCS) is an important evaluation index for assessing the mechanical properties of backfill materials, and it holds significant importance in the study of the applicability of mining backfill materials. Figure 7 shows the UCS of backfill samples in each group at different curing ages and the corresponding strength enhancement coefficients, where Figure 7a presents the UCS results and Figure 7b presents the calculated strength enhancement coefficients. As shown in Figure 7a, UCS of all groups increases with curing age, with a significant growth rate. Comparing the UCS of the backfill at the same curing age, the highest value at 7 days is observed in the A2 group, while the highest value at 28 days is found in the A1 group, indicating that the strength is optimal for each group at the corresponding curing age. Additionally, UCS of the high-PG cemented paste backfill in different ratios is above 0.5 MPa at 7 days and above 1 MPa at 28 days. Notably, the A2 group achieves a strength of over 0.8 MPa at 7 days and over 1.5 MPa at 28 days (the stress–strain curve and failure sample of the A2 group are shown in Figure 8). Compared with recent PG-based backfill systems that commonly rely on NaOH activation or alkaline washing to improve UCS [24,25], the tested 40 wt.% PG clinker-free multi-solid-waste binder met the UCS requirement for the targeted phosphate-mine backfill while improving PG utilization [26].

To quantitatively investigate the variation in UCS, the strength enhancement coefficient was introduced, and the calculated values are shown in Figure 7b. The expression for the strength enhancement coefficient is given in Equations (4) and (5) [15]:

(4) $$\lambda_{i}=\frac{{UCS}_{i,28}}{{UCS}_{i,7}}$$

(5) $$\xi_{i,t}=\frac{{UCS}_{i,28}}{{UCS}_{A1,t}}$$

where $UCS_{i,7}$ and $UCS_{i,28}$ are the measured uniaxial compressive strengths of mixture i at 7 and 28 days, respectively; $UCS_{i,t}$ is the measured UCS of mixture i at curing age t, and $UCS_{A1,t}$ is the measured UCS of the reference mixture A1 at the same curing age. Thus, $\lambda_{i}$ represents the age-related strength development of each mixture (*i* = 1, 2, 3, 4), while $\xi_{i,t}$ represents the relative strength of each mixture compared with A1 at the same curing age (*i* = 2, 3, 4). These coefficients were calculated directly from the UCS results shown in Figure 7a.

Using the strength enhancement coefficient *ξ* for UCS at the same curing age can explain the influence of different components on strength. If *ξ* is greater than 1, it indicates that the current mix shows better strength performance compared to the first group used as the initial condition. If *ξ* is less than 1, it indicates a weakening effect. The UCS enhancement coefficients *ξ* for each group are shown in Figure 7. From *ξ_i,7_*, it can be seen that at 7 days, the enhancement coefficient of A2 is greater than 1, while the enhancement coefficients of A2 and A3 are both less than 1, with the coefficients gradually decreasing. This suggests that in the early stage of curing, the hydration reaction in A2 is stronger than in A1, and the hydration products contribute more to the improvement in strength. From *ξ_i,28_*, it can be observed that at 28 days, the enhancement coefficients of A2–A4 are all less than 1, and the coefficients gradually decrease, indicating that the UCS of the A1–A4 groups gradually decreases. This is because groups with higher SSP content and lower activity exhibit insufficient hydration in the later stages, resulting in fewer hydration products and a weaker effect on pore structure optimization. Therefore, the strength variation trend differs from that at 7 days.

The use of the strength enhancement coefficient *λ* for each group at different curing ages allows for comparison of the strength enhancement capacity of different groups’ hydration reactions. If *λ* is greater than 1, it indicates an enhancing effect on the UCS; if *λ* is less than 1, it indicates a weakening effect. The UCS enhancement coefficient *λ* for each group is shown in Figure 7. From *λ_i_*, it can be seen that the strength enhancement coefficients of the A1–A4 groups are all significantly greater than 1, with an overall decreasing trend. Among them, A1 has the largest strength enhancement coefficient, with the 28-day strength being 2.331 times that of the 7-day strength, while A4 has the smallest, with the 28-day strength being 1.881 times that of the 7-day strength. This indicates that, after the hydration reaction, the UCS steadily increases, but the degree of increase is affected by the mix ratio. As the SSP content increases, the amount of active substances decreases, leading to weakened later-stage hydration reactions and a reduced filling effect on the pores. This result corresponds with the microstructural hydration mechanism analysis discussed earlier.

### 3.3. Cross-Scale Sensitivity Analysis

The analysis of the 7-day UCS of the backfill shows that the strength of the A2 group is superior to that of the A1 group, indicating that the hydration reaction in A2 may be stronger than in A1. This contrasts with the judgment that “the activity of GBFS is higher than that of SSP” [27]. To analyze the factors affecting the early 7-day strength, the discussion focuses on microstructure and cross-scale sensitivity analysis. Previous studies have shown that when the backfill is subjected to compressive failure, internal damage results from the coupling of initial pore damage and load-induced damage [19]. Therefore, a derivative analysis of the internal microstructure of the backfill at 7 days is conducted. The sensitivity of the pore structure to UCS is studied through porosity, pore probability entropy, and pore fractal dimension, and the influence patterns on the backfill strength are summarized from a cross-scale perspective.

**Figure 9:**
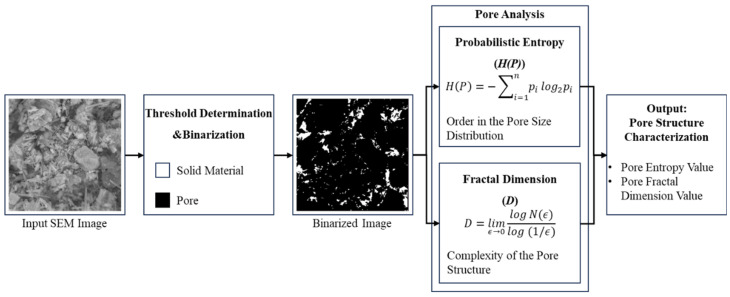
Probabilistic Entropy and Fractal Dimension Calculation Process.

The cross-scale sensitivity analysis focused on the 7-day results and used the measured UCS, NMR-derived porosity, and image-based pore parameters, including pore area probability entropy and fractal dimension. The representative SEM images were used to support the morphological interpretation of hydration products and pore filling, while the sensitivity analysis was based on extracted pore parameters rather than visual observation alone. Among the three microparameters, porosity directly represents the total pore area ratio within the backfill, and the specific data is obtained through inversion using NMR instrument. The pore area probability entropy and fractal dimension are derived from binarized SEM images using mathematical calculation software, with the calculation process shown in Figure 9.

The pore area probability entropy represents the degree of order in the pore size distribution, i.e., the randomness of the pore size distribution within the backfill. A lower entropy value indicates a more uniform pore size and more regular spatial distribution, while a higher entropy value suggests significant diversity in pore sizes and a stronger randomness in spatial distribution. The calculation formula is as follows [28]:

(6) $$H(P)=-\sum_{i=1}^{n}p_{i}{log}_{2}p_{i}$$

In the equation, *H(P)* represents the pore probability entropy; *n* denotes the total number of intervals into which the pore area is divided, and in this experiment, *n* is taken as 10; and *p_i_* represents the probability that the pore area falls into the i-th interval.

The fractal dimension is a key parameter for quantitatively describing the complexity of the pore structure in the backfill [29]. In this study, the box-counting method is used to calculate the fractal dimension of the pores. The space of the pore distribution is continuously divided into smaller boxes, and the number of boxes that contain at least one pore is counted. The relationship between the box size and the number of boxes is then fitted to obtain the fractal dimension, as shown in Equation (7) [15].

(7) $$D=\underset{\epsilon \to 0}{\lim}\frac{\log N(\epsilon )}{log(1/\epsilon )}$$

In the equation, *D* represents the fractal dimension of the pores, *ϵ* represents the box size, and *N*_(*ϵ*)_ represents the minimum number of boxes required to cover the pore distribution when the box size is *ϵ*.

In addition, to quantitatively analyze the relationship between the pore microparameters and the strength patterns, this study introduces the parameter sensitivity, as shown in the following formula [30]:

(8) $$S=\left(\frac{Y_{i}-Y_{1}}{Y_{1}}\right)/\left(\frac{P_{i}-P_{1}}{P_{1}}\right)$$

In the equation, *S* represents the parameter sensitivity, *Y_i_* represents the microparameter of the varying group (*i* = 2, 3, 4), Y_1_ represents the microparameter of the first group as the reference group, *P_i_* represents UCS of the varying group (*i* = 2, 3, 4), and *P*_1_ represents the UCS of the first group as the reference group.

The microparameters and their sensitivity at 7 days of curing for the backfill are shown in Table 5.

As shown in Table 5, the sensitivity (*S*) of the three selected parameters exhibits the same trend across different groups, indicating a stable correspondence between these three parameters and the uniaxial compressive strength (UCS) of the high-PG cemented paste backfill. To reduce the experimental single-value error, the average sensitivity is used. The absolute values of the microparameter sensitivities are categorized into three levels for analysis: generally significant (0 < |s¯| < 0.5), significant (0.5 < |s¯| < 1), and highly significant (|s¯| > 1).

As shown in Table 5, a stable negative correlation exists between porosity and UCS, with an average sensitivity of −0.547, indicating a significant impact on UCS. This suggests that as the amount of active substances in the cementitious material decreases, the hydration products reduce, leading to incomplete internal structure and an increase in the total pore area ratio, which directly affects the physical properties of the PG-based cemented paste backfill.

As shown in Table 5, the calculated range of pore area probability entropy is [0.323, 0.497], indicating that the pore area distribution of the cemented paste backfill generally shows a concentration trend with good uniformity. Furthermore, when the cementitious material contains more active substances, the hydration products can better encapsulate the aggregates, resulting in a more concentrated pore size distribution. Additionally, there is a stable negative correlation between pore area probability entropy and UCS, with an average sensitivity of −2.009, indicating a highly significant impact on UCS. This suggests that pore area probability entropy is an important parameter for assessing the macroscopic strength properties of High-PG cemented paste backfill, and a uniform pore size distribution contributes to strength enhancement.

As shown in Table 5, the calculated range of fractal dimension for the high-PG cemented paste backfill is [1.478, 1.614], indicating that the pore complexity of the High-PG cemented paste backfill at 7 days is at a moderate level, with pores relatively well-defined along the particle boundaries [31,32]. When the cementitious material contains more active substances, the fractal dimension decreases, resulting in smaller pore spacing and a simpler pore structure. Additionally, there is a stable negative correlation between fractal dimension and UCS, with an average sensitivity of −0.454, indicating a generally significant effect on UCS. Therefore, the variation in fractal dimension has a negative correlation with UCS, but it is less sensitive compared to other microparameters.

In conclusion, the pore structure of the high-PG cemented paste backfill at 7 days is well-developed, with a relatively concentrated pore size distribution. Porosity, pore area probability entropy, and fractal dimension all exhibit a stable negative correlation with UCS, indicating that the internal pore structure directly affects the backfill strength. The complexity of the pores, pore size distribution, and pore area ratio all have a direct impact on the backfill strength, with the pore size distribution having the most significant effect. Combining Figure 5 with the porosity data, it is clear that at 7 days, the porosity of A_2_ is lower than that of A1, but the *T*_2_ main peak of A_2_ is more concentrated between 0.1–10 ms, indicating that the pore distribution of A2 is more focused on micropores. Therefore, the development mechanism of the 7-day strength can be summarized as follows: the early strength is not solely determined by the amount of active material. The initial hydration reaction relies on SO_4_^2−^ and alkalinity provided by PG and SSP, allowing AFt and C–S–H to play a “framework-filling” role, refining the pore size and suppressing the formation of large interconnected pores. Among the groups, the A2 group (with 30% SSP and GBFS content) shows the best match. The cross-scale results were interpreted in an age-dependent manner. At 7 days, A1 and A2 showed close NMR pore characteristics, but A2 had a higher UCS, indicating that early strength was affected not only by total porosity but also by pore distribution and local microstructural arrangement. At 28 days, A1 showed lower NMR porosity and the highest UCS, suggesting more favorable later-age densification. Therefore, A2 was regarded as the mixture with better early-age performance, while A1 showed better later-age pore refinement and strength development.

## 4. Conclusions

This study investigated a clinker-free multi-solid-waste binder containing 40 wt.% PG for cemented paste backfill by combining UCS testing, XRD, SEM, NMR, and pore-structure sensitivity analysis. Compared with many reported PG-based backfill systems that use lower PG dosages, cement/lime addition, alkaline washing, or strong alkali activation, the present study focused on evaluating whether PG can be incorporated at a relatively high dosage while still meeting the strength requirement of the targeted phosphate-mine backfill. The main conclusions are as follows:

(1) In the high-PG cemented paste backfill, PG supplied abundant SO_4_^2−^, while SSP and GBFS provided Ca-bearing and aluminosilicate components for hydration reactions. The formation of AFt and C–S–H-related gel products contributed to a framework-filling structure, which was associated with pore refinement and strength development.
(2) The pore structure of the high-PG cemented paste backfill is well-developed. Porosity, pore area probability entropy, and fractal dimension all exhibit a stable negative correlation with UCS, indicating that pore complexity, pore size distribution, and pore area ratio directly affect the backfill strength, with pore size distribution having the most significant impact on the 7-day strength. In particular, the sensitivity analysis of the 7-day strength with respect to microstructural parameters confirms that, in the high-PG binder system, the combined effect of different constituents on pore refinement plays a more dominant role in strength development than the total amount of reactive substances.
(3) The UCS results show that the SSP/GBFS ratio controlled the age-dependent strength development of the 40 wt.% PG binder. The A2 mixture, containing 30 wt.% SSP and 30 wt.% GBFS, achieved the highest 7-day UCS of 0.8 MPa, whereas the A1 mixture, containing 25 wt.% SSP and 35 wt.% GBFS, showed the highest 28-day UCS of 1.6 MPa. This indicates that a balanced SSP/GBFS ratio benefits early strength, while a higher GBFS proportion favors later-age strength. The tested PG clinker-free multi-solid-waste formulation therefore met the cemented backfill strength requirement for the targeted phosphate mine while improving PG utilization.

## Figures and Tables

**Table 1:** Particle size distribution of tailings.

Particle Size (μm)	<74	74–150	150–350	300–600	600–1180	1180–2360	2360–4750
Volume content (%)	10.11	5.91	6.34	11.91	18.05	31.97	15.71
Cumulative volume (%)	10.11	16.02	22.36	34.27	52.32	84.29	100

**Table 2:** Particle size distribution of slime.

Particle Size (μm)	<5	5–10	10–20	20–40	40–80	80–160	160–320
Volume content (%)	20.88	13.78	15.92	20.43	22.18	6.68	0.13
Cumulative volume (%)	20.88	34.66	50.58	71.01	93.19	99.87	100

**Table 3:** Chemical composition of cementitious materials.

Chemical Composition	SiO_2_	CaO	Al_2_O_3_	MgO	MnO	SO_3_	Fe_2_O_3_	F	P_2_O_5_	Others
SSP (%)	31.84	52.42	1.54	3.17	1.61	-	0.75	-	0.01	8.66
GBFS (%)	31.46	31.31	19.22	10.74	1.29	-	0.68	-	-	5.30
PG (%)	9.39	36.36	1.30	-	-	46.75	-	1.39	1.48	1.94

**Table 4:** Ratio of SSP to GBFS in Cementitious Materials.

Group	SSP (%)	GBFS (%)
A1	25	35
A2	30	30
A3	35	25
A4	40	20

**Table 5:** Microscopic parameters and sensitivity analysis results of backfill.

Parameter	Microparameter Value		UCS (MPa)		Sensitivity	
	Varying Group (*Y_i_*)	Reference Group (*Y*_1_)	Varying Group (*Y_i_*)	Reference Group (*Y*_1_)		
Porosity	7.618	8.380	0.800	0.695	−0.602	−0.547
	8.669		0.655		−0.599	
	8.671		0.640		−0.439	
Probability entropy	0.323	0.415	0.800	0.695	−1.467	−2.009
	0.497		0.655		−3.433	
	0.452		0.640		−1.127	
Fractal dimension	1.478	1.497	0.800	0.695	−0.084	−0.454
	1.522		0.655		−0.290	
	1.614		0.640		−0.988	

## Data Availability

The original contributions presented in this study are included in the article. Further inquiries can be directed to the corresponding author.
